# Association of β2-adrenergic receptor gene polymorphisms (rs1042713, rs1042714, rs1042711) with asthma risk: a systematic review and updated meta-analysis

**DOI:** 10.1186/s12890-019-0962-z

**Published:** 2019-11-07

**Authors:** Songlin Zhao, Wei Zhang, Xiuhong Nie

**Affiliations:** Department of Respiratory, Xuanwu Hospital Capital Medical University, No. 45, Changchun Street, Xicheng District, Beijing, 100053 China

**Keywords:** ADRB2, Polymorphism, Asthma, Meta-analysis

## Abstract

**Background:**

The published data on the association between β2-adrenergic receptor gene polymorphisms and asthma susceptibility are inconclusive. To derive a more precise estimation of this association, a meta-analysis was performed.

**Methods:**

A literature search was conducted in PubMed, Web of Science, EMBASE, Wanfang, and the China National Knowledge Infrastructure (CNKI) databases to identify eligible studies. The pooled odds ratios (ORs) with corresponding 95% confidence intervals (CIs) were used to calculate the strength of the association. A sensitivity analysis was performed to evaluate the influence of individual studies on the overall effect estimates, and funnel plots and Egger’s tests were used for indications of publication bias.

**Results:**

Seventy three studies with three single nucleotide polymorphisms (SNP) (rs1042713, c.G46A, p.Gly16Arg; rs1042714, c.G79C, p.Gln27Glu; rs1042711, c.T-47C, p.Cys19Arg) were finally identified. For the rs1042713 polymorphism, no significant association with asthma risk was found in the overall population. However, a significant protective association was found in the Indian population in the dominant model comparison (OR = 0.72, 95% CI = 0.59–0.87, I^2^ = 25%, studies = 5, cases = 1190, controls = 1241). A significant risk association was found in the Arab population in the dominant model comparison (OR = 1.75, 95% CI = 1.14–2.70, I^2^ = 0%, studies = 2, cases = 307, controls = 361) and the homozygote model comparison (OR = 1.88, 95% CI = 1.17–3.02, I^2^ = 0%, studies = 2, cases = 307, controls = 361), and in the Hispanic-Latino population in the dominant model comparison (OR = 1.68, 95% CI = 1.10–2.55, I^2^ = 77%, studies = 5, cases = 1026, controls = 1412). For the rs1042714 polymorphism, we found a significant association in the recessive model comparison (OR = 0.83, 95% CI = 0.70–0.98, I^2^ = 44%, studies = 52, cases = 8242, controls = 16,832), the homozygote genotype comparison (OR = 0.84, 95% CI = 0.72–0.98, I^2^ = 25%, studies = 52, cases = 8242, controls = 16,832) and the allelic genetic model (OR = 0.91, 95% CI = 0.83–0.99, I^2^ = 59%, studies = 52, cases = 8242, controls = 16,832) in the overall population. When stratified by age, a significant association was also found in children in the recessive model comparison (OR = 0.59, 95% CI = 0.39–0.88, I^2^ = 58%, studies = 18, cases = 2498, controls = 2510) and the homozygote genotype comparison (OR = 0.63, 95% CI = 0.43–0.92, I^2^ = 46%, studies = 18, cases = 2498, controls = 2510), but not in adult. For the rs1042711 polymorphism, no significant associations were found in the any genetic model.

**Conclusion:**

The meta-analysis suggests that the ADRB2 rs1042714 polymorphism has a protective association with asthma in the overall population and the pediatric subgroup.

## Background

Asthma is a chronic respiratory inflammation disease characterized by airway hyperresponsiveness, reversible airway obstruction and airway wall remodeling [1]. It is believed to be a multifactorial disorder with a strong genetic component in its pathogenesis [2, 3]. So far, many studies have evaluated the association between genetic variants and asthma susceptibility. Numerous genes have been identified as asthma-susceptible genes, from which the β-2 adrenergic receptor (ADRB2) is the most widely studied [4–6].

ADRB2 is encoded by an intronless gene on chromosome 5q31, which is abundantly expressed on many airway cells including smooth muscle cells [7, 8]. ADRB2 transcript has a 5′ leader cistron (5′ LC) harboring a short open reading frame (ORF) that encodes a 19-amino acid peptide, which regulates mRNA translation and controls the cellular expression of ADRB2. A variation at position 19 that causes a change from cysteine (Cys) to arginine (Arg) was reported in the 5′ LC, and this variation plays a role in regulating ADRB2 gene expression [9–11]. However, little is known regarding the possible role of this polymorphism in asthma. In addition, two missense variations (rs1042713, c.G46A, p.Gly16Arg and rs1042714, c.G79C, p.Gln27Glu) that occur in high allelic frequency in the general population have been identified, corresponding to a change from glycine (Gly) to arginine (Arg) at amino acid position 16 and glutamate (Gln) to glutamine (Glu) at amino acid position 27 [12]. Studies in vitro [13] and primary cultures of cells expressing these endogenous variants [14] illustrated the different phenotypes between the polymorphic receptors. The Gly16 receptor could enhance agonist-promoted downregulation of receptor expression compared with the Arg16 receptor. In contrast, the Glu27 receptor is relatively resistant to agonist-promoted downregulation compared with the Gln27 receptor [13, 14]. Genetic studies have indicated that these variations not only affect the risk of asthma, but also affect the therapeutic outcomes of inhaled β2-adrenergic receptor agonists [15–20].

Considering the impact of the asthma risk potentially resulting from ADRB2 gene variations, a number of case-control studies have explored the association between the ADRB2 gene polymorphisms and asthma risk in different ethnicities [21–23]. However, these results are conflicting and inconclusive, which are possibly due to the limitations associated with an individual studies and small sample size. To shed light on these contradictory results and to more precisely evaluate the relationship between the ADRB2 gene polymorphisms and asthma risk, several meta-analyses concerning the association between ADRB2 gene polymorphisms and asthma have been reported [15, 24–29]. However, these meta-analyses have also shown inconsistent results. After publication of these meta-analyses, many additional case-control studies about the ADRB2 polymorphisms on asthma risk were carried out [30–34]. Therefore, we present the results of a comprehensively updated meta-analysis of all relevant published data to investigate the associations between ADRB2 gene polymorphisms and asthma risk with a focus on rs1042713, rs1042714 and rs1042711 polymorphisms.

## Methods

This systematic review was conducted in accordance with the Preferred Reporting Items for Systematic Reviews and Meta-Analyses (PRISMA) Statement guidelines.

### Publication search

Publications were obtained from the PubMed, EMBASE, Web of Science, the Chinese National Knowledge Infrastructure, and Wanfang databases (the last search was conducted on September 1, 2018). The keywords searched in our investigation were (asthma or asthmatic) and (β2-adrenergic receptor or ADRB2 or β2-AR or beta2-adrenoreceptor or β2-adrenoceptor) and (polymorphism or mutation or variant or rs1042713 or G46A or Gly16Arg or rs1042714 or G79C or Gln27Glu or rs1042711 or T-47C or Cys19Arg). The search was performed in duplicate by two independent reviewers (Songlin Zhao and Wei Zhang).

### Inclusion and exclusion criteria

The inclusion criteria of our study were as follows: (1) any human studies that estimated the prevalence of the β2-adrenergic receptor polymorphisms and asthma risk were included, which were published in English and Chinese. (2) They were case-control studies. (3) The genotype distributions or allele frequency of each study should be available for estimating an odds ratio with a 95% confidence interval. (4) When eligible papers had insufficient information, we contacted the authors for additional information via email. Studies were excluded from our meta-analysis if their authors did not provide us with the related data.

### Data extraction

The basic information extracted from each study was as follows: name of the first author, publication year, country, ethnicity, age of cases and controls, sample size, and genotype frequencies in cases and controls. The data were extracted independently and in duplicate by two reviewers (Songlin Zhao and Wei Zhang) who used a standardized data extraction form. Any disagreement was adjudicated by a third author (Xiuhong Nie).

### Study quality assessment and meta-analysis quality assessment

The Newcastle-Ottawa Scale (NOS) was used to assess the quality of the included studies. The items assessed included selection, comparability of case/controls, exposure/outcome, age and gender. The quality scores ranged from 0 to 9. We divided the NOS scores into three levels (higher quality, score ≥ 7; moderate quality, 4 ≤ score < 7; low quality, score < 4).

A Measurement Tool to Assess Systematic Reviews 2 (AMSTAR 2) was used to assess the quality of the systematic reviews [35]. The AMSTAR 2 calculator queries 16 items of relevance that provide insight into the quality of the systematic review methodology.

### Statistical analysis

A Hardy-Weinberg equilibrium (HWE) was assessed for each study by use of the Pearson’s chi-square test in control groups, and significance was set at *P* < 0.05. The pooled ORs for the ADRB2 polymorphisms and asthma risk were calculated for the dominant genetic model, the recessive genetic model, the homozygote genetic model and the allele genetic model. The heterogeneity was assessed by using the Q-test and I^2^ test. A *P*-value> 0.10 of Q-test and I^2^ < 50% indicated a lack of heterogeneity among the studies; then, the fixed-effect model was used. Otherwise, the random effect model was used. Subgroup analyses were performed regarding ethnicity, case age and HWE *P*-value. The ethnicity subgroups were designed as Caucasian, Hispanic-Latinos, non-Hispanic Blacks, East-Asian, Indian and Arab. Age subgroups were designed as Adult and pediatric subgroups. HWE *P*-value subgroups were designed as *P*-value> 0.05 and *P*-value< 0.05 subgroups. Sensitivity analysis was conducted by sequentially excluding one study at a time to examine the effect of each study on the combined result. The funnel plot and Egger’s test was used to assess the potential publication bias. All the statistical analyses of this meta-analysis were performed using the STATA 11.0 software (State Corporation, College Station, TX, USA).

## Results

Characteristics of the studies included in the meta-analysis.

**Fig. 1:**
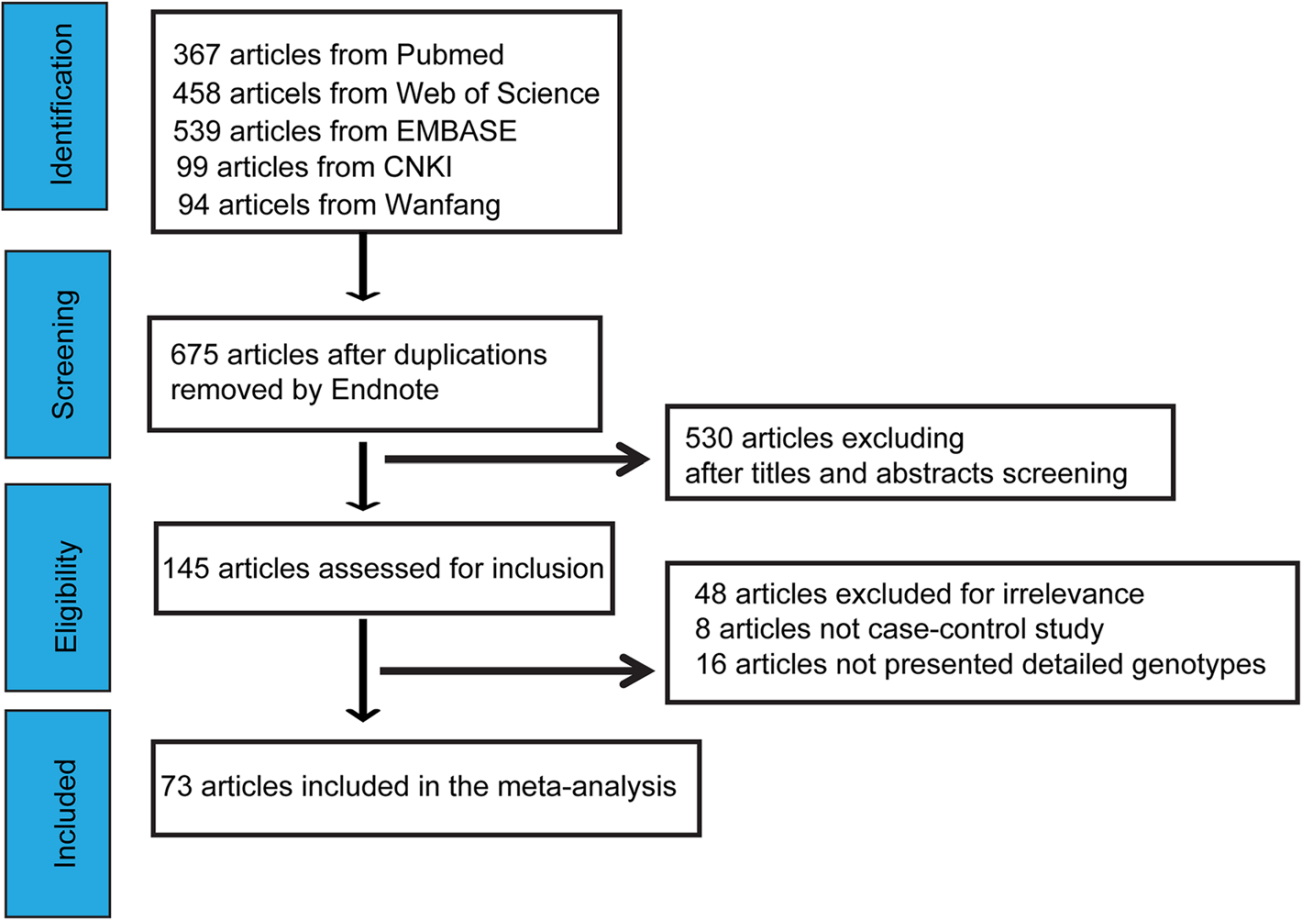
The flow diagram of inclusion of studies in the meta-analysis. The selection pipeline for the series is indicated including the comprehensive search criteria, screening process and details of the eligible studies

The flow chart in Fig. 1 outlines the study selection process. After a comprehensive search of the PubMed, Web of Science, EMBASE, CNKI and Wanfang databases, a total of 1557 articles were identified. First, we excluded 882 duplicated studies. After reading the abstracts and titles, 530 studies were excluded. The remaining 145 studies were then assessed for inclusion. Of these, 72 studies were excluded because 48 studies were not irrelevance, 16 studies lacked detailed genotypes, and 8 studies lacked a case-control design. Finally, a total of 73 studies met the inclusion criteria and were included in the meta-analysis [8, 12, 17, 21–23, 30–34, 36–97]. The characteristics of each eligible study are shown in Additional file 1. Of these 73 studies, each contains two independent studies, and the data is extracted accordingly. During the process, if the sum of genotype distribution of one study is over than 1, the study is excluded from the meta-analysis. The genotype, allele distribution and *P*-value of HWE for the rs1042713, rs1042714 and rs1042711 polymorphisms are respectively shown in Additional files 2, 3 and 4.

### Meta-analysis of ADRB2 rs1042713 polymorphism and asthma risk

**Fig. 2:**
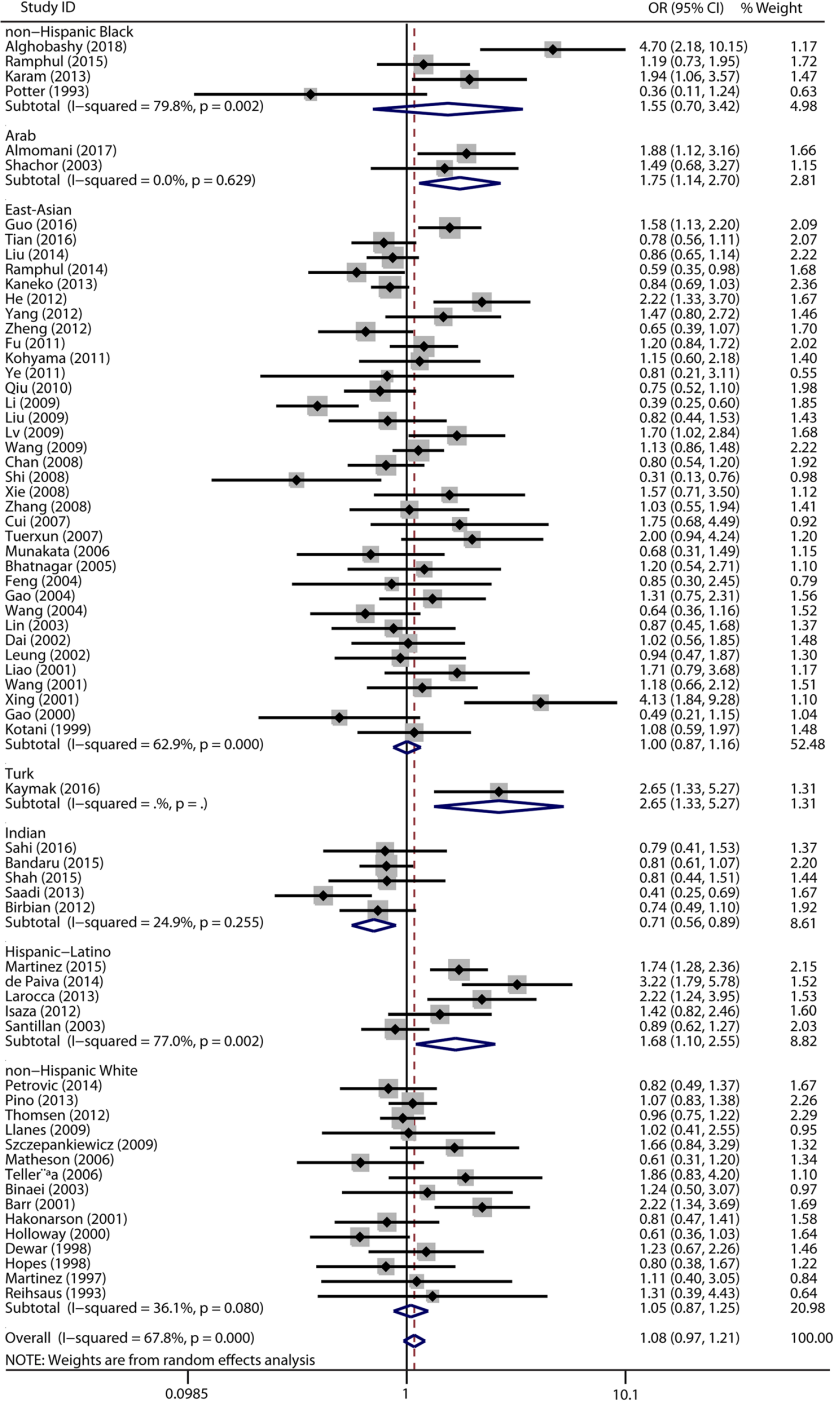
Forest plots of the association between the ADRB2 rs1042713 polymorphism and risk of asthma in dominant model comparison. GG + GA vs. AA genotype. Each study is shown by an OR estimate with the corresponding 95% CIs. The horizontal lines denote the 95% CIs and the squares represent the point OR estimate of each study. The size of the square is proportional to its inverse-variance weight in the meta-analysis. The diamond represents the pooled meta-analysis effect size estimate. The stratified meta-analysis was performed regarding the ethnicity

Among the 73 publications included in the meta-analysis, 71 studies investigated the association between the ADRB2 rs1042713 polymorphism and asthma risk (Additional file 2). Overall, there was no significant association in any of the genetic model comparisons in the total population (Table 1, Additional file 5: Figure S1, Additional file 6: Figure S2, Additional file 7: Figure S3 and Fig. 2). In the stratified analysis of ethnicity, a significant protective association was found in the Indian population in the dominant model comparison (OR = 0.72, 95% CI = 0.59–0.87, I^2^ = 25%, studies = 5, cases = 1190, controls = 1241), and a significant risk association was found in the Arab population in the dominant model comparison (OR = 1.75, 95% CI = 1.14–2.70, I^2^ = 0%, studies = 2, cases = 307, controls = 361) and the homozygote model comparison (OR = 1.88, 95% CI = 1.17–3.02, I^2^ = 0%, studies = 2, cases = 307, controls = 361) (Table 1, Fig. 2). In addition, we found a significant association in the Hispanic-Latino population in the dominant model comparison (OR = 1.68, 95% CI = 1.10–2.55, I^2^ = 77%, studies = 5, cases = 1026, controls = 1412) (Table 1, Fig. 2), consistent with a previous study [25]. No associations were found in adults and children regarding age or the *P*-value of HWE (Table 1).

**Table 1:** Results of the pooled and subgroup analyses for the ADRB2 rs1042713 polymorphism and asthma risk

Variable	n	Case/Control	Dominant model comparison			Recessive model comparison			Homozygote genotype comparison			Allelic comparison			Frequency of minor allele (A/(A + G))	
			OR[95%CI]	*P*_(Z)_	I^2^	OR[95%CI]	*P*_(Z)_	I^2^	OR[95%CI]	*P*_(Z)_	I^2^	OR[95%CI]	*P*_(Z)_	I^2^	Case	Control
Total	71	12,070/21669	1.08 [0.97, 1.21]	0.165	68%	1.05 [0.94, 1.18]	0.387	70%	1.10 [0.95, 1.27]	0.201	70%	1.02 [0.95, 1.10]	0.591	74%	0.494	0.443
Adult	36	6984/15697	1.12 [0.97, 1.28]	0.122	62%	1.05 [0.92, 1.19]	0.447	58%	1.12 [0.96, 1.32]	0.160	56%	1.06 [0.98, 1.15]	0.131	56%	0.485	0.430
Children	26	4539/5320	1.13 [0.93, 1.38]	0.210	72%	1.07 [0.85, 1.35]	0.570	79%	1.13 [0.85, 1.51]	0.400	79%	1.00 [0.85, 1.17]	0.976	84%	0.510	0.478
East-Asian	36	6343/6291	1.00 [0.87, 1.16]	0.966	63%	1.04 [0.87, 1.26]	0.645	71%	1.03 [0.84, 1.26]	0.801	67%	1.02 [0.93, 1.13]	0.677	69%	0.539	0.533
Indian	5	1190/1241	0.72 [0.59, 0.87]	0.001	25%	0.91 [0.69, 1.19]	0.480	54%	0.72 [0.51, 1.03]	0.070	53%	0.84 [0.69, 1.02]	0.073	60%	0.477	0.437
Arab	2	307/361	1.75 [1.14, 2.70]	0.011	0%	1.17 [0.59, 2.30]	0.654	69%	1.88 [1.17, 3.02]	0.009	35%	0.83 [0.34, 2.02]	0.165	51%	0.360	0.436
Hispanic-Latinos	5	1026/1412	1.68 [1.10, 2.55]	0.016	77%	1.64 [0.86, 3.14]	0.133	90%	2.09 [0.98, 4.45]	0.055	64%	0.99 [0.60, 1.63]	0.970	94%	0.464	0.481
non-Hispanic Blacks	5	671/556	1.55 [0.70, 3.42]	0.282	80%	1.22 [0.68, 2.19]	0.498	52%	1.40 [0.64, 3.06]	0.401	64%	0.70 [0.55, 0.89]	0.529	85%	0.529	0.532
Caucasian	16	2892/11950	1.05 [0.87, 1.25]	0.620	36%	0.96 [0.83, 1.11]	0.541	35%	1.01 [0.87, 1.16]	0.946	32%	0.99 [0.89, 1.10]	0.847	46%	0.430	0.389
HWE(*P* > 0.05)	49	9752/19296	0.99 [0.87, 1.13]	0.890	70%	0.97 [0.85, 1.10]	0.598	73%	0.98 [0.81, 1.16]	0.716	76%	0.98 [0.90, 1.07]	0.653	78%	0.486	0.431
HWE(*P* < 0.05)	18	2366/2320	1.22 [0.96, 1.55]	0.240	55%	1.17 [0.94, 1.44]	0.164	50%	1.17 [0.99, 1.38]	0.055	29%	1.15 [1.01, 1.30]	0.031	48%	0.520	0.541

### Meta-analysis of the ADRB2 rs1042714 polymorphism and asthma risk

**Fig. 3:**
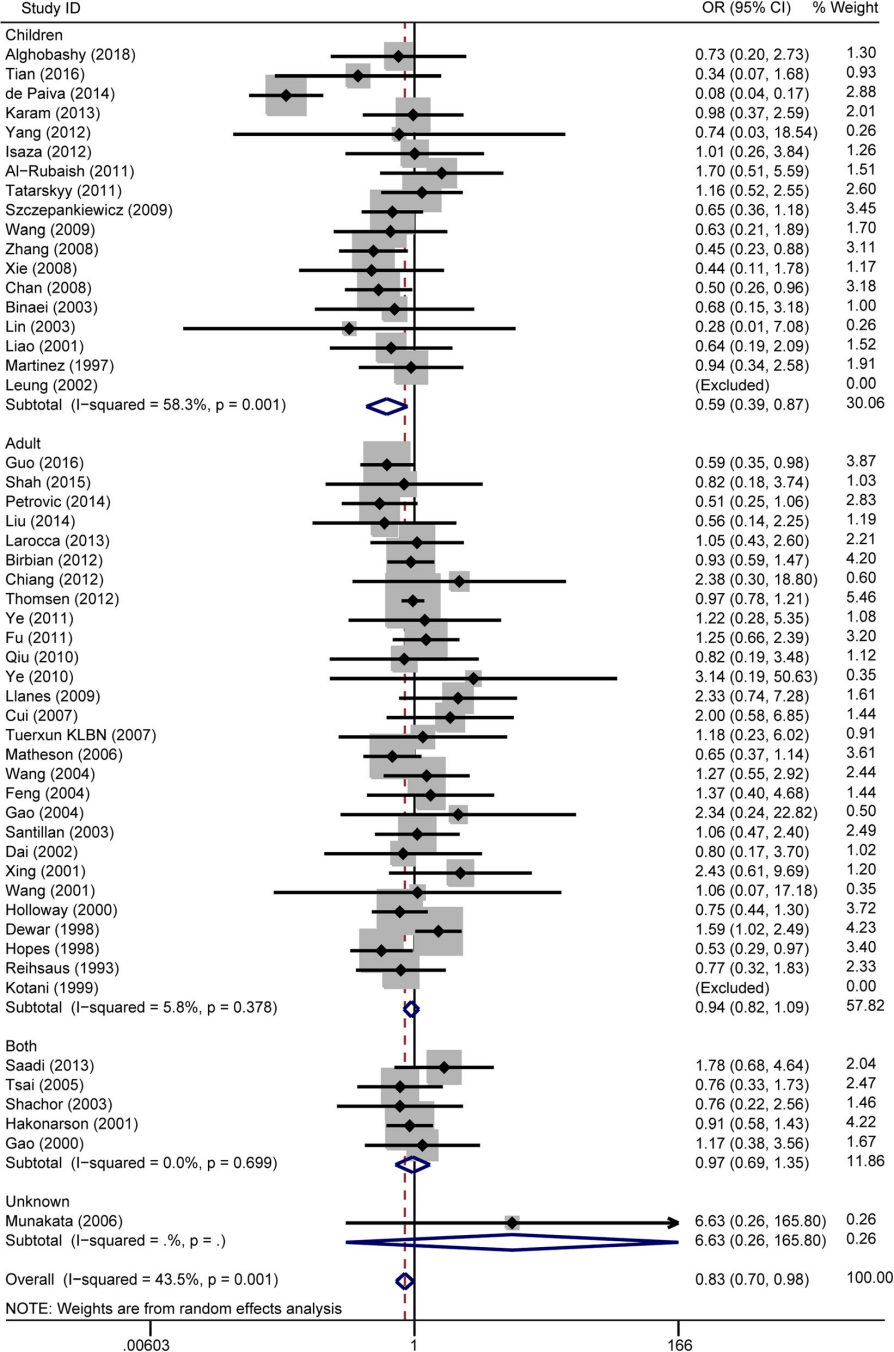
Forest plots of the association between the ADRB2 rs1042714 polymorphism and risk of asthma in recessive model comparison. GG vs. CC + CG genotype. Each study is shown by an OR estimate with the corresponding 95% CIs. The horizontal lines denote the 95% CIs and the squares represent the point OR estimate of each study. The size of the square is proportional to its inverse-variance weight in the meta-analysis. The diamond represents the pooled meta-analysis effect size estimate. The stratified meta-analysis was performed regarding the age

**Fig. 4:**
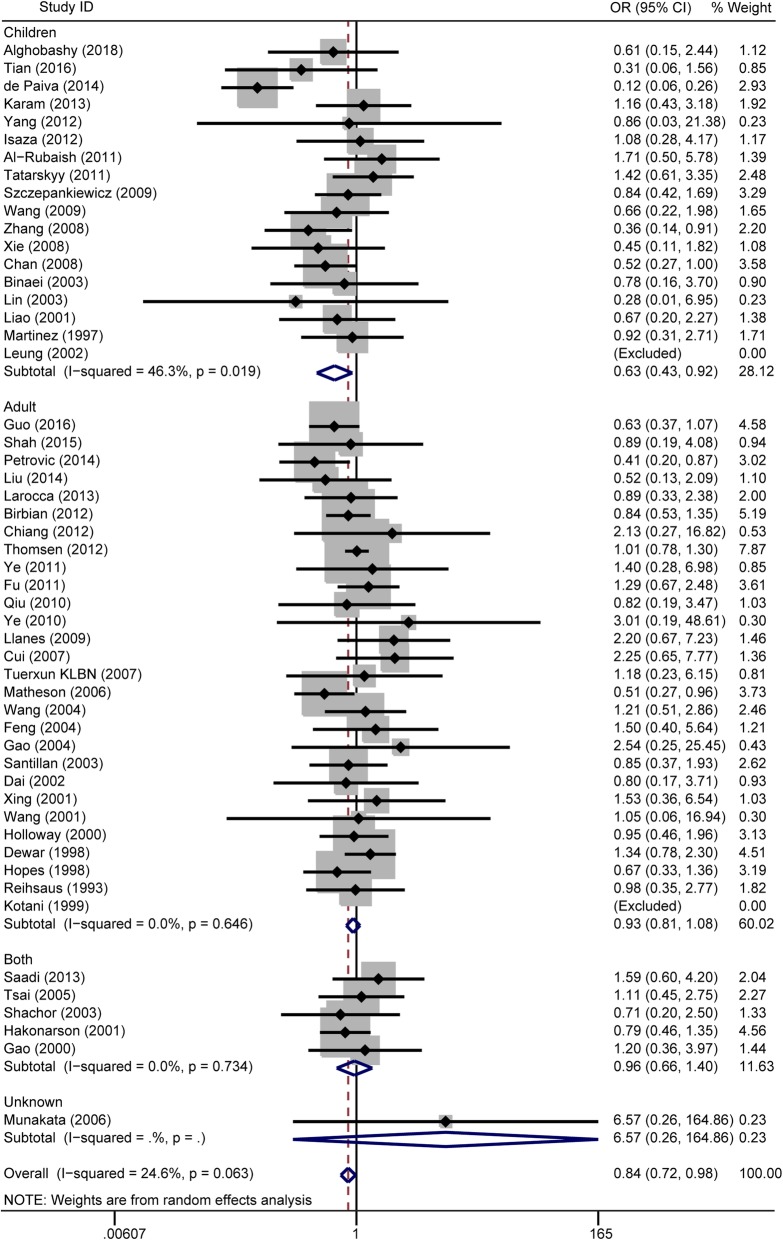
Forest plots of the association between the ADRB2 rs1042714 polymorphism and risk of asthma in homozygote genotype comparison. GG vs. CC genotype. Each study is shown by an OR estimate with the corresponding 95% CIs. The horizontal lines denote the 95% CIs and the squares represent the point OR estimate of each study. The size of the square is proportional to its inverse-variance weight in the meta-analysis. The diamond represents the pooled meta-analysis effect size estimate. The stratified meta-analysis was performed regarding the age

There were 52 studies investigating the association between the rs1042714 polymorphism and asthma risk (Additional file 3). In the overall population, significant associations were found in the recessive model comparison (OR = 0.83, 95% CI = 0.70–0.98, I^2^ = 44%, studies = 52, case = 8242, control = 16,832) (Fig. 3), the homozygote genotype comparison (OR = 0.84, 95% CI = 0.72–0.98, I^2^ = 25%, studies = 52, cases = 8242, controls = 16,832) (Fig. 4) and the allelic genetic model (OR = 0.91, 95% CI = 0.83–0.99, I^2^ = 59%, studies = 52, cases = 8242, controls = 16,832) (Additional file 8: Figure S4), but not in the dominant model comparison (Additional file 9: Figure S5 and Table 2). When stratified by age, significant associations were also found in children in the recessive model comparison (OR = 0.59, 95% CI = 0.39–0.88, I^2^ = 58%, studies = 18, cases = 2498, controls = 2510) (Fig. 3) and the homozygote genotype comparison (OR = 0.63, 95% CI = 0.43–0.92, I^2^ = 46%, studies = 18, cases = 2498, controls = 2510) (Fig. 4) but not in adults (Table 2). In the analysis stratified by ethnicity, no associations were found in any ethnic group. In the analysis stratified by the *P*-value of HWE, significant associations were found in the *P* > 0.05 subgroup in the dominant model comparison (OR = 0.89, 95% CI = 0.80–0.99, I^2^ = 32%, studies = 35, cases = 5552, controls = 14,306), and the allelic genetic model (OR = 0.91, 95% CI = 0.84–0.99, I^2^ = 28%, studies = 35, cases = 5552, controls = 14,306). No associations were found in the *P* < 0.05 subgroup (Table 2).

**Table 2:** Results of the pooled and subgroup analyses for the ADRB2 rs1042714 polymorphism and asthma risk

Variable	n	Case/Control	Dominant model comparison			Recessive model comparison			Homozygote genotype comparison			Allelic comparison			Frequence of minor allele (G/(G + C))	
			OR[95%CI]	*P*_(Z)_	I^2^	OR[95%CI]	*P*_(Z)_	I^2^	OR[95%CI]	*P*_(Z)_	I^2^	OR[95%CI]	*P*_(Z)_	I^2^	Case	Control
Total	52	8242/16832	0.92 [0.83, 1.02]	0.097	45%	0.83 [0.70, 0.98]	0.032	44%	0.84 [0.72, 0.98]	0.028	25%	0.91 [0.83, 0.99]	0.029	59%	0.242	0.348
Adult	28	5022/13609	0.89 [0.77, 1.02]	0.101	55%	0.94 [0.83, 1.07]	0.372	6%	0.94 [0.81, 1.08]	0.371	0%	0.92 [0.83, 1.02]	0.095	52%	0.246	0.371
Children	18	2498/2510	0.97 [0.81, 1.16]	0.706	36%	0.59 [0.39, 0.88]	0.009	58%	0.63 [0.43, 0.92]	0.016	46%	0.86 [0.69, 1.07]	0.169	73%	0.205	0.234
East-Asian	27	4555/4217	0.94 [0.85, 1.04]	0.249	20%	0.81 [0.65, 1.01]	0.057	0%	0.82 [0.66, 1.03]	0.090	0%	0.93 [0.85, 1.01]	0.085	25%	0.164	0.161
Indian	3	672/687	0.85 [0.68, 1.07]	0.160	18%	1.04 [0.70, 1.54]	0.862	0%	0.95 [0.63, 1.43]	0.806	0%	0.91 [0.76, 1.09]	0.294	0%	0.234	0.250
Arab	2	137/196	0.98 [0.63, 1.51]	0.249	0%	1.14 [0.49, 2.61]	0.764	0%	1.11 [0.47, 2.60]	0.810	0%	1.01 [0.71, 1.43]	0.972	0%	0.270	0.273
Hispanic-Latinos	4	605/982	0.67 [0.41, 1.10]	0.108	75%	0.54 [0.13, 2.25]	0.394	90%	0.54 [0.18, 1.64]	0.276	82%	0.61 [0.33, 1.15]	0.129	91%	0.202	0.277
non-Hispanic Blacks	2	194/162	1.06 [0.53, 2.09]	0.875	58%	0.88 [0.40, 1.94]	0.758	0%	0.94 [0.41, 2.12]	0.876	0%	1.03 [0.74, 1.45]	0.845	41%	0.309	0.290
Caucasian	14	2079/10588	0.98 [0.81, 1.19]	0.840	51%	0.90 [0.78, 1.03]	0.126	29%	0.92 [0.79, 1.08]	0.319	11%	0.94 [0.82, 1.07]	0.338	50%	0.418	0.437
HWE(*P* > 0.05)	35	5552/14306	0.89 [0.80, 0.99]	0.034	32%	0.94 [0.83, 1.06]	0.299	0%	0.94 [0.81, 1.08]	0.358	0%	0.91 [0.84, 0.99]	0.021	28%	0.258	0.366
HWE(*P* < 0.05)	16	2478/2474	0.95 [0.77, 1.17]	0.637	59%	0.70 [0.47, 1.06]	0.089	69%	0.73 [0.51, 1.05]	0.088	58%	0.87 [0.70, 1.09]	0.239	79%	0.221	0.248

### Meta-analysis of the ADRB2 rs1042711 polymorphism and asthma risk

For the rs1042711 polymorphism, only seven case-control studies provided genotype distribution data (Additional file 4); therefore, no subgroup analysis was conducted. No significant associations were found in the overall population in any of the genetic models. The results are shown in Table 3.

**Table 3:** Results of the pooled analyses for the ADRB2 rs1042711 polymorphism and asthma risk

Items	Number		
Study	7		
Case	1769		
Control	1941		
	OR[95%CI]	*P*_(Z)_	I^2^
Dominant model comparison	0.91 [0.73, 1.14]	0.433	53%
Recessive model comparison	1.24 [0.89, 1.74]	0.200	3%
Homozygote genotype comparison	1.21 [0.86, 1.71]	0.271	9%
Allelic comparison	0.95 [0.78, 1.18]	0.665	61%
Frequence of minor allele (C/(T + C))			
Case	0.170		
Control	0.170		

### Publication bias

**Fig. 5:**
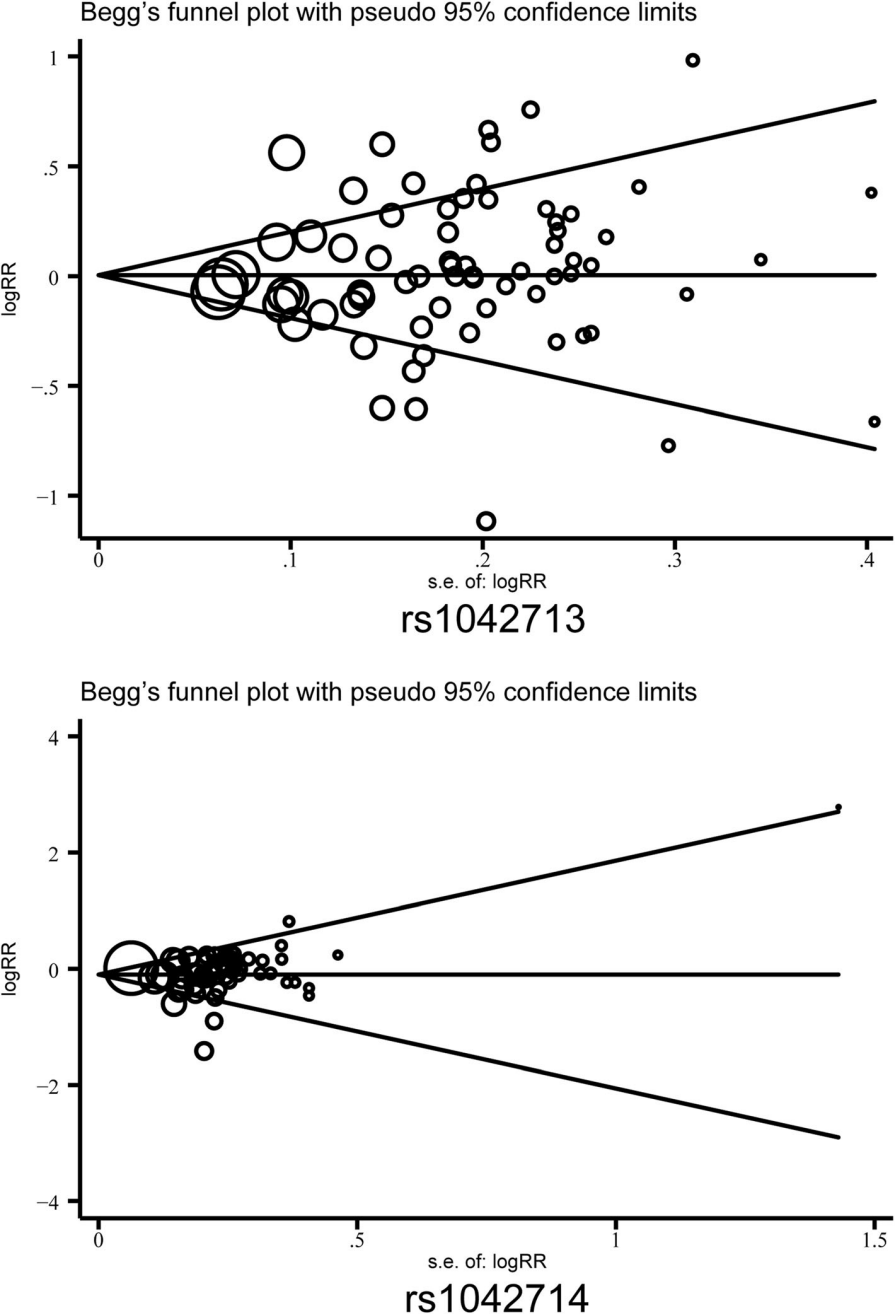
Begg’s funnel plot for publication bias on asthma susceptibility under the allele genetic model. The effect size (OR) was plotted on the y-axis, and the inverse of variance of the effect was plotted on the x-axis

Potential publication bias was investigated using the funnel plot and was further assessed using the Egger’s test. The shape of the funnel plot (Fig. 5) and the Egger’s test (Table 4) did not indicate any evidence of publication bias for the rs1042713, rs1042714 and rs1042711 polymorphisms in any of the genetic model comparisons.

**Table 4:** Publication bias results of Egger’s test

SNP	Study number	Dominant model comparison		Recessive model comparison		Homozygote genotype comparison		Allelic comparison	
		t	p	t	p	t	p	t	p
rs1042713	71	0.520	0.603	0.250	0.801	0.430	0.665	0.460	0.650
rs1042714	52	1.300	0.200	0.270	0.791	0.460	0.646	0.510	0.609
rs1042711	7	−0.110	0.916	−2.460	0.058	−2.560	0.050	−0.520	0.622

### Sensitivity analysis

**Fig. 6:**
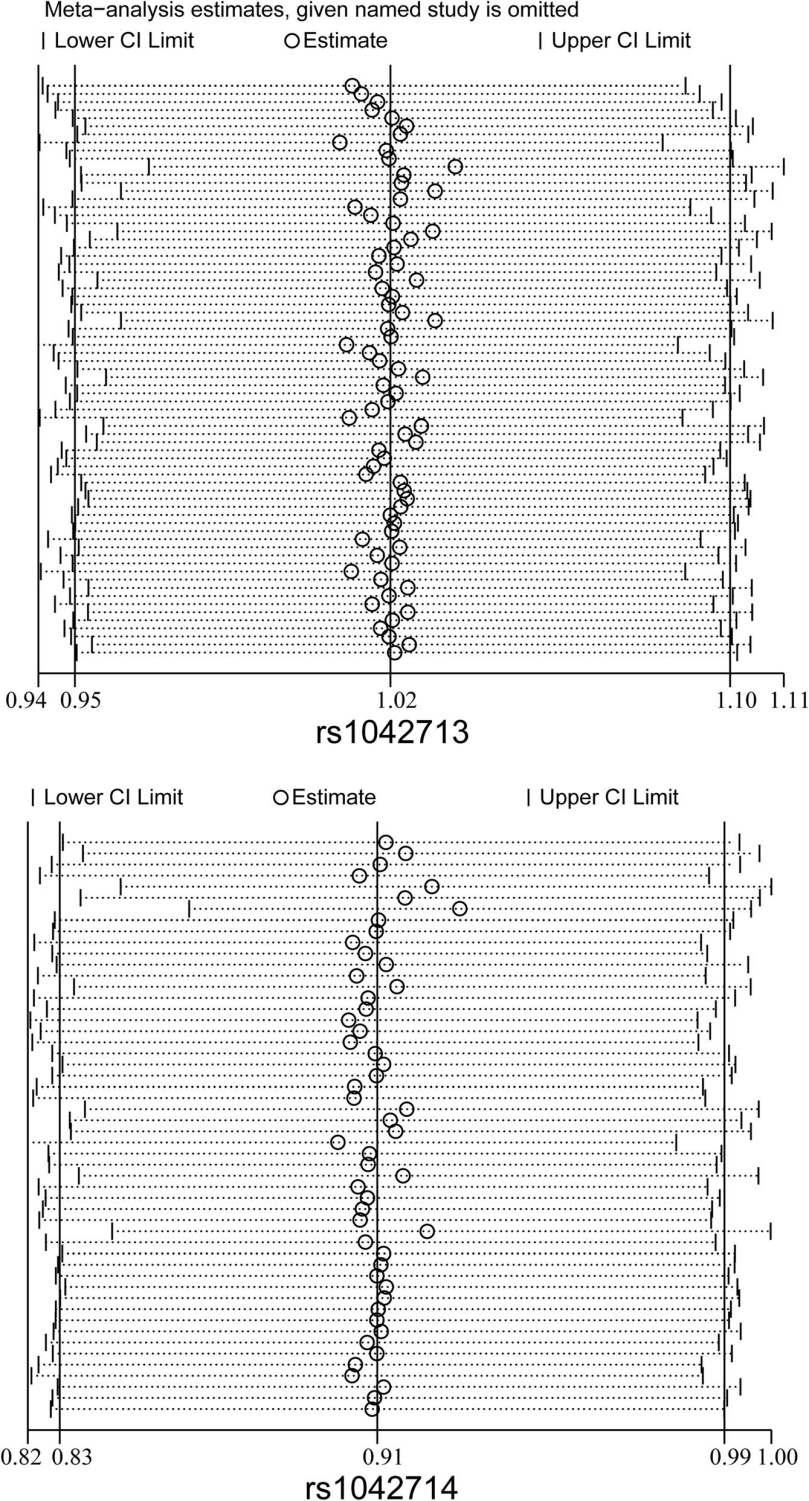
Sensitivity analysis for the ADRB2 rs1042713 (up) and rs1042714 (down) polymorphisms with risk of asthma under the allele genetic model. The combined result that omit one study is shown by an OR estimate with the corresponding 95% CIs

The sensitivity analysis was conducted by sequentially excluding individual studies to estimate the stability of the results. After sequentially excluding each study, similar statistically results were found (Fig. 6).

## Discussion

In this study, the associations between three ADRB2 gene SNPs (rs1042713, rs1042714 and rs1042711) and the risk of asthma were conducted base on the data from 73 studies involving 13,493 asthmatic patients and 22,931 controls. This meta-analysis showed that the rs1042713 polymorphism was not a risk factor for overall asthma susceptibility, which was consistent with most of the previous meta-analyses [15, 16, 25, 27–29]; however, the data contradict one of the latest meta-analyses [24]. The difference between these results is seemingly due to the different inclusion criteria. The inclusion criteria in the Xie et al. [24] meta-analysis used *P*-value of the HWE; however, when stratified by the *P*-value of the HWE, there was still no association between the Gly16Arg polymorphism and asthma in the HWE (*P* > 0.05) subgroup in our study and in that of Liang et al. [25]. When reviewing Xie et al.’s literature list, we found that they missed some studies that satisfied their inclusion criteria [31, 54, 77–79, 87, 88, 91, 92, 95], which may be a reason for the discrepancies.

Some studies reported that the genotype frequency and allele frequency of the rs1042713 polymorphism vary among different ethnic groups [98, 99]. A search of 1000 Genomes Project or Hapmap data showed an approximately 15% difference in allele frequency for the rs1042713 polymorphism. The Gly16 homozygous genotype frequency is more common in the non-Hispanic White than Chinese and more frequent in non-Hispanic White compared to non-Hispanic Black. Therefore, there is high heterogeneity for the rs1042713 polymorphism in the overall analysis. Even though we excluded some studies that did not meet the HWE, heterogeneity was not decreased. Selective bias in the literature has an important effect on the results of the overall meta-analysis. Large sample sizes can better reflect the truth of the effects of the rs1042713 polymorphism on the asthma risk.

Because the genotype frequency and allele frequency of the rs1042713 polymorphism vary among the different ethnic groups, we divided the ethnic groups according to the studies reported by Ortega et al. [100, 101], such that (1) non-Hispanic Whites of European ancestry were designated as Caucasian, (2) Mexicans and South Americans were designated as Hispanic-Latinos, (3) African Americans and non-Hispanic Blacks from Europe and Africa were designated as non-Hispanic Blacks; and (4) Chinese, Japanese and Korean individuals were considered separate Asian ethnic groups and designated as the East-Asian subgroup. In addition, Indian and Arab descendants were designated as separate ethnic groups because these ethnic subgroups have different genotypes and allele frequencies [98]. The analysis stratified by ethnicity showed a significant risk association in Hispanic-Latinos in the dominant model comparison, consistent with a previous study where a significant association in the South American population was found [25]. In addition, the previous study claimed to divide the cohorts by ethnicity, but for the most part, the cohorts were divided by continent when they combined Indian, Arab, Japanese and Han Chinese individuals as the Asian population [24, 25]. These populations have different genotypic and allelic frequencies for the rs1042713 polymorphism [98]. After the stratified analysis, the heterogeneities in the Arab and Indian population were decreased, and a protective association in the Indian population in the dominant model comparison and a risk association in the Arab population in the dominant model comparison and the homozygote genotype comparison were found. However, there is a need for further studies with larger sample sets.

For the rs1042714 polymorphism in the current meta-analysis, benefitting from the inclusion of more case-control studies, a protective effect was found not only in the pediatric subgroup in the recessive model comparison and the homozygote genotype comparison but also in the overall population in the recessive model comparison, the homozygote genotype comparison and the allelic genetic model. This was consistent with previous reports by Liang et al. [25] and Ammarin et al. [27] that showed a protective effect in the pediatric subgroup in the recessive model comparison and the homozygote genotype comparison, confirming that the Glu27 polymorphism was a protective effect for asthma. A genetic study showed that Glu27 homozygotes had less reactive airways than Gln27 homozygotes, and these results could further suggest a protective role for the Glu27 polymorphism in asthma [102]. In addition, in vitro and ex vivo functional studies indicated that Glu27 allele enhanced resistance to agonist-induced down regulation of the receptor, suggesting a protective role of Glu27 polymorphism in regard to receptor desensitisation [13, 14].

In the analysis stratified by HWE according to the *P*-value for the rs1042714 polymorphism, significant associations were found in the subgroup with *P* > 0.05 in the dominant model comparison and the allelic genetic model but not in the *P* < 0.05 subgroup. These results need to be interpreted with caution. The reason the control group population was not in HWE may be selection for a particular phenotype or that the population was not sufficiently large or random.

For the rs1042711 polymorphism, no significant associations with the risk of asthma were found in any comparison model. More research is needed because only seven case-controls were included in this study. There might not be sufficient statistical evidence to clarify the association between the rs1042711 polymorphism and the risk of asthma.

There could be several potential limitations to this meta-analysis. The first problem relates to the limitations of the literature. All available literature should be included in the meta-analysis, but we only included literature published in English and Chinese, thus neglecting studies published in other languages. Second, even though the existing literature had acceptable quality, detailed information was not provided such as asthma definition varied among different articles and this may be a confounding factor. Using a self- or physician-diagnosis of asthma can be confounded by individuals who do not have asthma such as older subjects with a smoking history who could have COPD. These physician-diagnosed cohorts many times do not have an objective diagnostic basis of asthma based on methacholine BHR or beta agonist responsiveness which could result in confounded and undetected associations. In addition, the participation rates for cases and controls were not reported in the majorities of included studies; thus, our meta-analysis was unable to explore the selection bias. Moreover, with limited information about maternal constitutional and environmental risk factors for asthma (such as smoking history), we could not evaluate the gene-gene and gene-environmental interactions. The different definitions of asthma and the environmental factors in individual studies were obvious sources of clinical heterogeneity and may produce bias. Therefore, moderate-to-high heterogeneities were found in some genetic models for the Gly16Arg polymorphism. Stratification by ethnicity may help to improve homogeneity among studies, but it may also influence statistical power. In addition, some meta-analysis studies claimed to divide the cohorts by ethnicity but it seems like the cohorts were actually being divided by continent for the most part, it will induce the contradictory findings. Third, the Gly16Arg and Gln27Glu polymorphisms tagging for rare variants modulated therapeutic responses and contributed to asthma risk [103]; however, these variants were not specifically genotyped.

## Conclusion

In conclusion, the present meta-analysis indicates that the rs1042714 polymorphism is an important genetic protective factor to decrease the risk of developing asthma, especially in children. The rs1042713 polymorphism may be involved in the risk of asthma in the Arab and Hispanic-Latino populations and a protective factor in the Indian population. However, more well-designed and high-quality studies with larger sample sizes should be conducted to support this finding in various ethnic groups.

## Supplementary information

**Additional file 1: Table S1.** Characteristics of the studies included in the meta-analysis. (XLSX 14 kb)

**Additional file 2: Table S2**. Genotype and allele distribution in the meta-analysis for the rs1042713 polymorphism. (XLSX 17 kb)

**Additional file 3: Table S3**. Genotype and allele distribution in the meta-analysis for the rs1042714 polymorphism. (XLSX 15 kb)

**Additional file 4: Table S4.** Genotype and allele distribution in the meta-analysis for the rs1042711 polymorphism. (XLSX 9 kb)

**Additional file 5: Figure S1.** Forest plots of the association between the ADRB2 rs1042713 polymorphism and risk of asthma in recessive model comparison. GG vs. GA + AA genotype. Each study is shown by an OR estimate with the corresponding 95% CIs. The horizontal lines denote the 95% CIs and the squares represent the point OR estimate of each study. The size of the square is proportional to its inverse-variance weight in the meta-analysis. The diamond represents the pooled meta-analysis effect size estimate. The stratified meta-analysis was performed regarding the ethnicity. (TIF 19900 kb)

**Additional file 6: Figure S2.** Forest plots of the association between the ADRB2 rs1042713 polymorphism and risk of asthma in homozygote genotype comparison. GG vs. AA genotype. Each study is shown by an OR estimate with the corresponding 95% CIs. The horizontal lines denote the 95% CIs and the squares represent the point OR estimate of each study. The size of the square is proportional to its inverse-variance weight in the meta-analysis. The diamond represents the pooled meta-analysis effect size estimate. The stratified meta-analysis was performed regarding the ethnicity. (TIF 19918 kb)

**Additional file 7: Figure S3.** Forest plots of the association between the ADRB2 rs1042713 polymorphism and risk of asthma in the allele comparison. G vs. A allele. Each study is shown by an OR estimate with the corresponding 95% CIs. The horizontal lines denote the 95% CIs and the squares represent the point OR estimate of each study. The size of the square is proportional to its inverse-variance weight in the meta-analysis. The diamond represents the pooled meta-analysis effect size estimate. The stratified meta-analysis was performed regarding the ethnicity. (TIF 19763 kb)

**Additional file 8: Figure S4.** Forest plots of the association between the ADRB2 rs1042714 polymorphism and risk of asthma in the allele comparison. G vs. C allele. Each study is shown by an OR estimate with the corresponding 95% CIs. The horizontal lines denote the 95% CIs and the squares represent the point OR estimate of each study. The size of the square is proportional to its inverse-variance weight in the meta-analysis. The diamond represents the pooled meta-analysis effect size estimate. The stratified meta-analysis was performed regarding the ethnicity. (TIF 23295 kb)

**Additional file 9: Figure S5.** Forest plots of the association between the ADRB2 rs1042714 polymorphism and risk of asthma in dominant model comparison. GG + CG vs. CC genotype. Each study is shown by an OR estimate with the corresponding 95% CIs. The horizontal lines denote the 95% CIs and the squares represent the point OR estimate of each study. The size of the square is proportional to its inverse-variance weight in the meta-analysis. The diamond represents the pooled meta-analysis effect size estimate. The stratified meta-analysis was performed regarding the age. (TIF 24131 kb)

## Data Availability

The datasets used and/or analyzed during the current study are available from the corresponding author on reasonable request.

## Abbreviations

ADRB2: β2-adrenergic receptor
AMSTAR: A Measurement Tool to Assess Systematic Reviews
CI: Confidence intervals
CNKI: China National Knowledge Infrastructure
HWE: Hardy-Weinberg equilibrium
NOS: Newcastle-Ottawa Scale
OR: Odds ratios
PRISMA: Preferred Reporting Items for Systematic Reviews and Meta-Analyses
SNP: Single nucleotide polymorphism
