# Supplementary figures and images for: Association of β2-adrenergic receptor gene polymorphisms (rs1042713, rs1042714, rs1042711) with asthma risk: a systematic review and updated meta-analysis
Source: BMC Pulm Med. 2019 Nov 7;19:202. doi: 10.1186/s12890-019-0962-z (PMC6836544; doi:10.1186/s12890-019-0962-z)

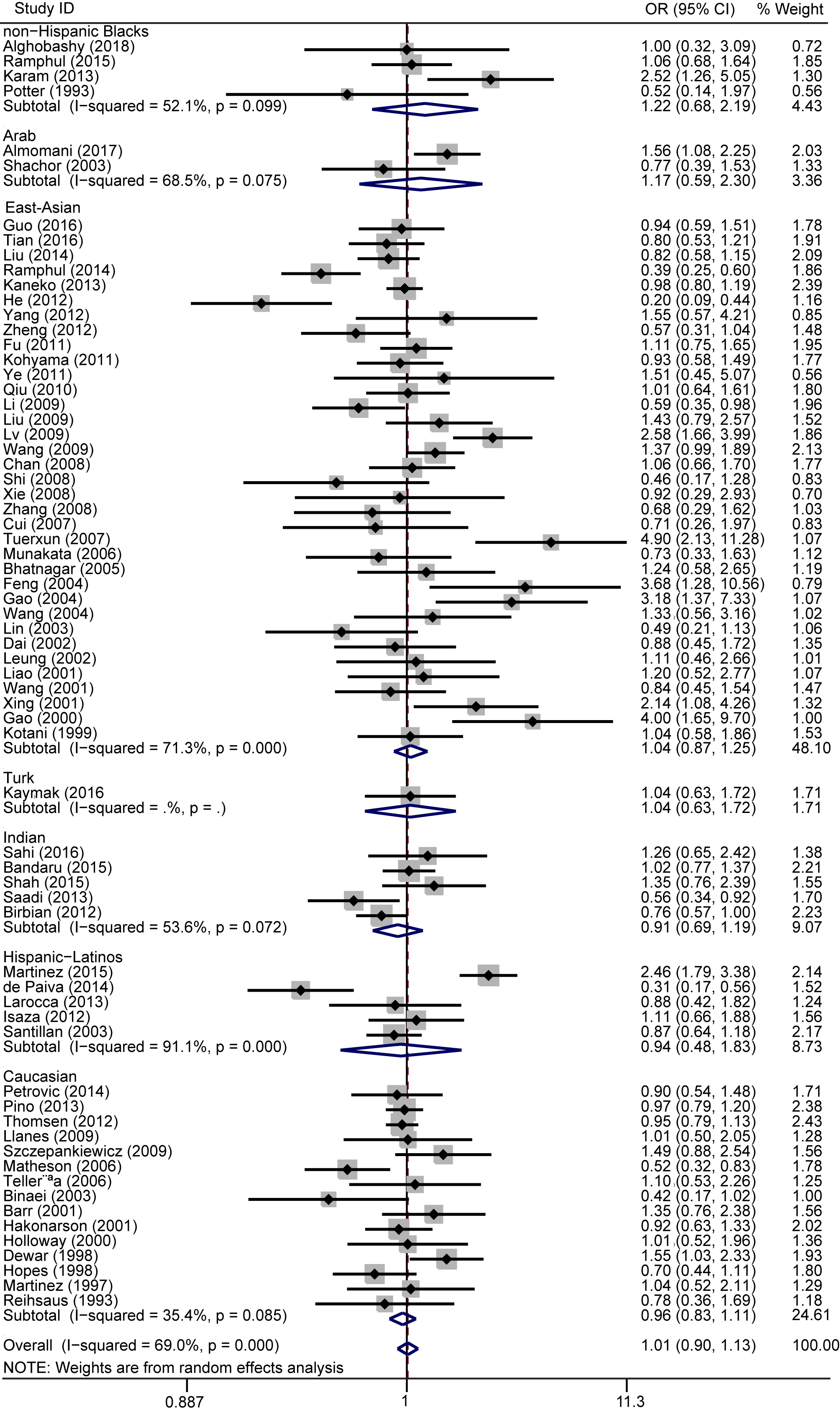

Supplement: Supplementary file 5 — Additional file 5: Figure S1. Forest plots of the association between the ADRB2 rs1042713 polymorphism and risk of asthma in recessive model comparison. GG vs. GA + AA genotype. Each study is shown by an OR estimate with the corresponding 95% CIs. The horizontal lines denote the 95% CIs and the squares represent the point OR estimate of each study. The size of the square is proportional to its inverse-variance weight in the meta-analysis. The diamond represents the pooled meta-analysis effect size estimate. The stratified meta-analysis was performed regarding the ethnicity. (TIF 19900 kb) [file 12890_2019_962_MOESM5_ESM.tif]

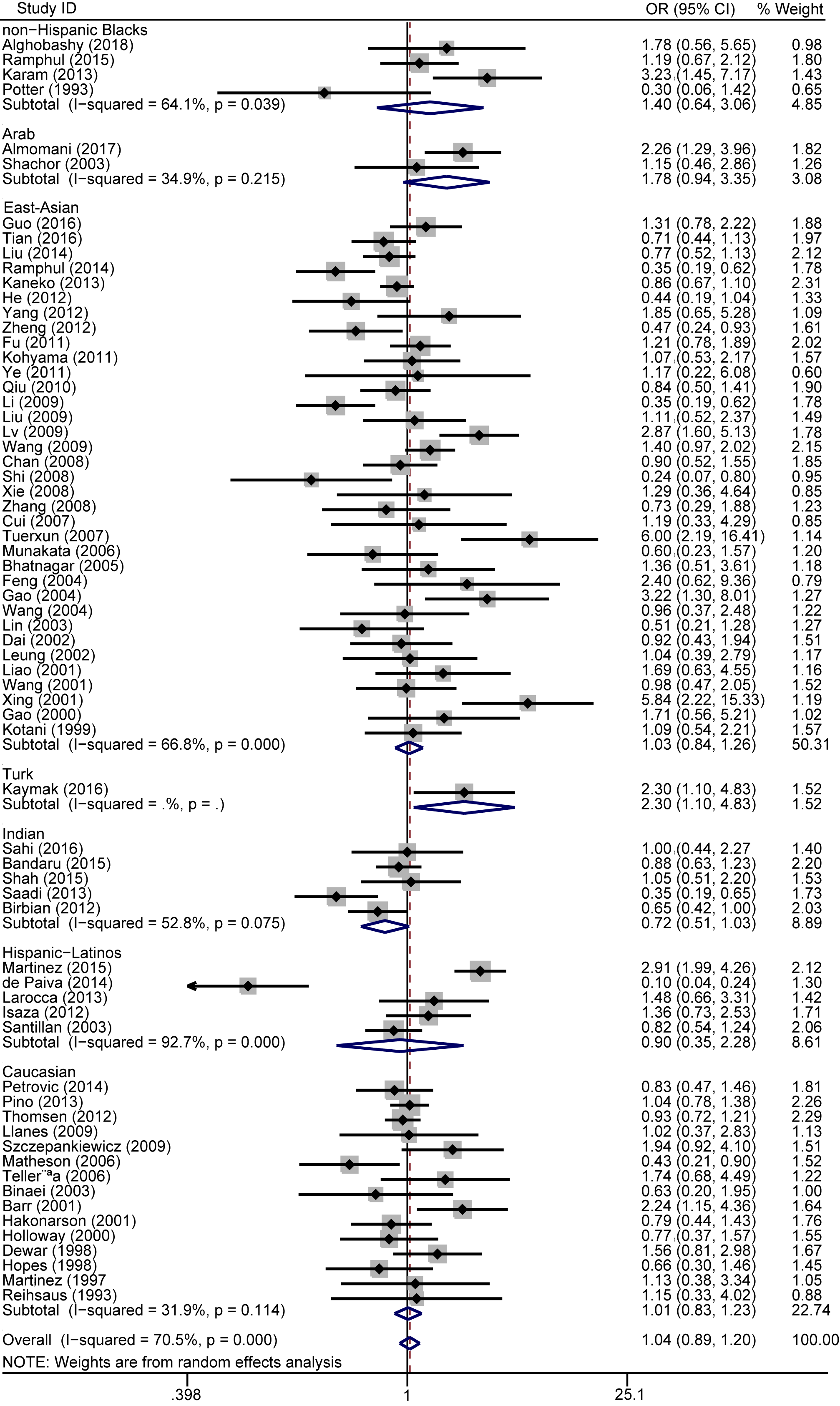

Supplement: Supplementary file 6 — Additional file 6: Figure S2. Forest plots of the association between the ADRB2 rs1042713 polymorphism and risk of asthma in homozygote genotype comparison. GG vs. AA genotype. Each study is shown by an OR estimate with the corresponding 95% CIs. The horizontal lines denote the 95% CIs and the squares represent the point OR estimate of each study. The size of the square is proportional to its inverse-variance weight in the meta-analysis. The diamond represents the pooled meta-analysis effect size estimate. The stratified meta-analysis was performed regarding the ethnicity. (TIF 19918 kb) [file 12890_2019_962_MOESM6_ESM.tif]

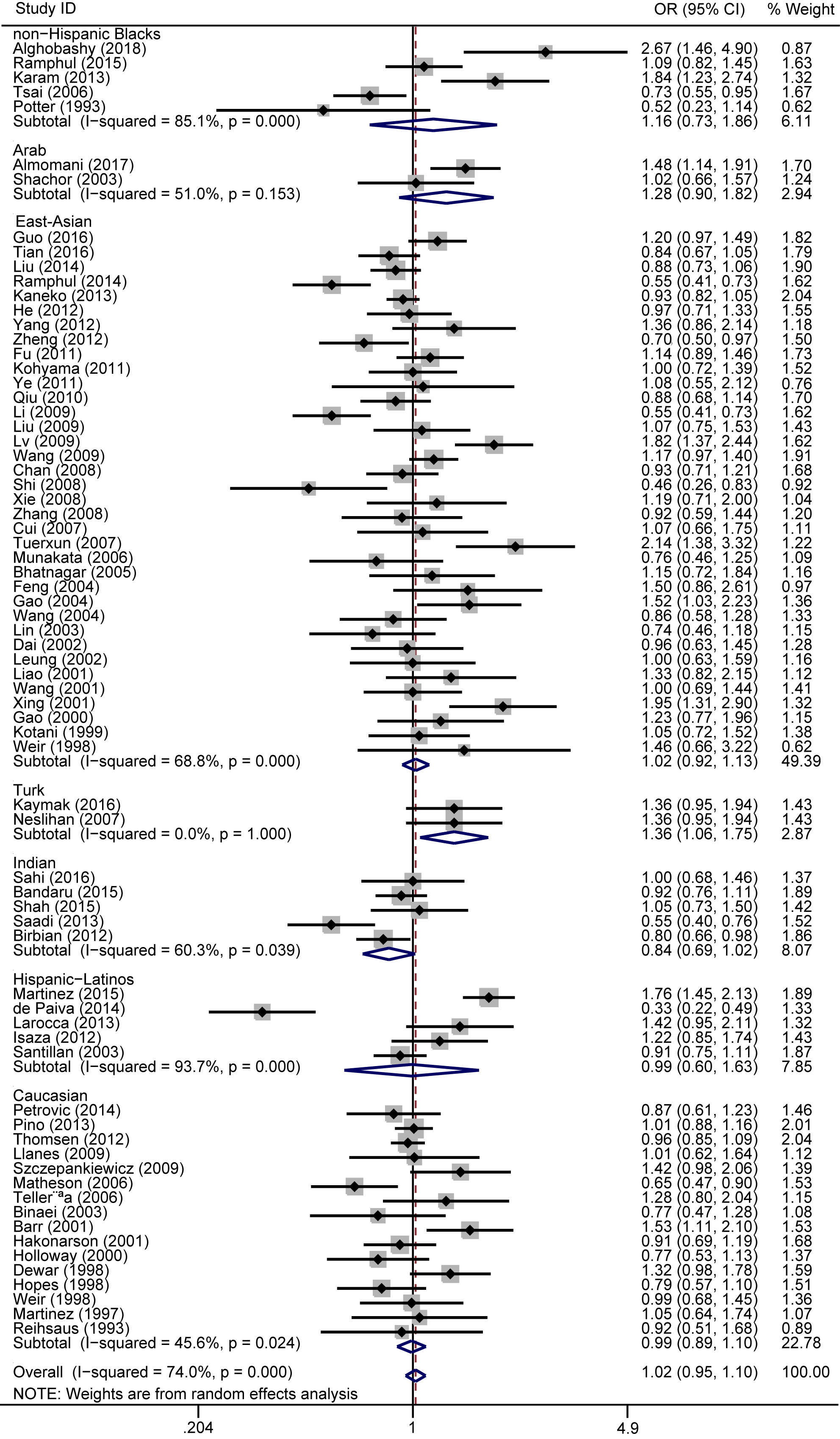

Supplement: Supplementary file 7 — Additional file 7: Figure S3. Forest plots of the association between the ADRB2 rs1042713 polymorphism and risk of asthma in the allele comparison. G vs. A allele. Each study is shown by an OR estimate with the corresponding 95% CIs. The horizontal lines denote the 95% CIs and the squares represent the point OR estimate of each study. The size of the square is proportional to its inverse-variance weight in the meta-analysis. The diamond represents the pooled meta-analysis effect size estimate. The stratified meta-analysis was performed regarding the ethnicity. (TIF 19763 kb) [file 12890_2019_962_MOESM7_ESM.tif]

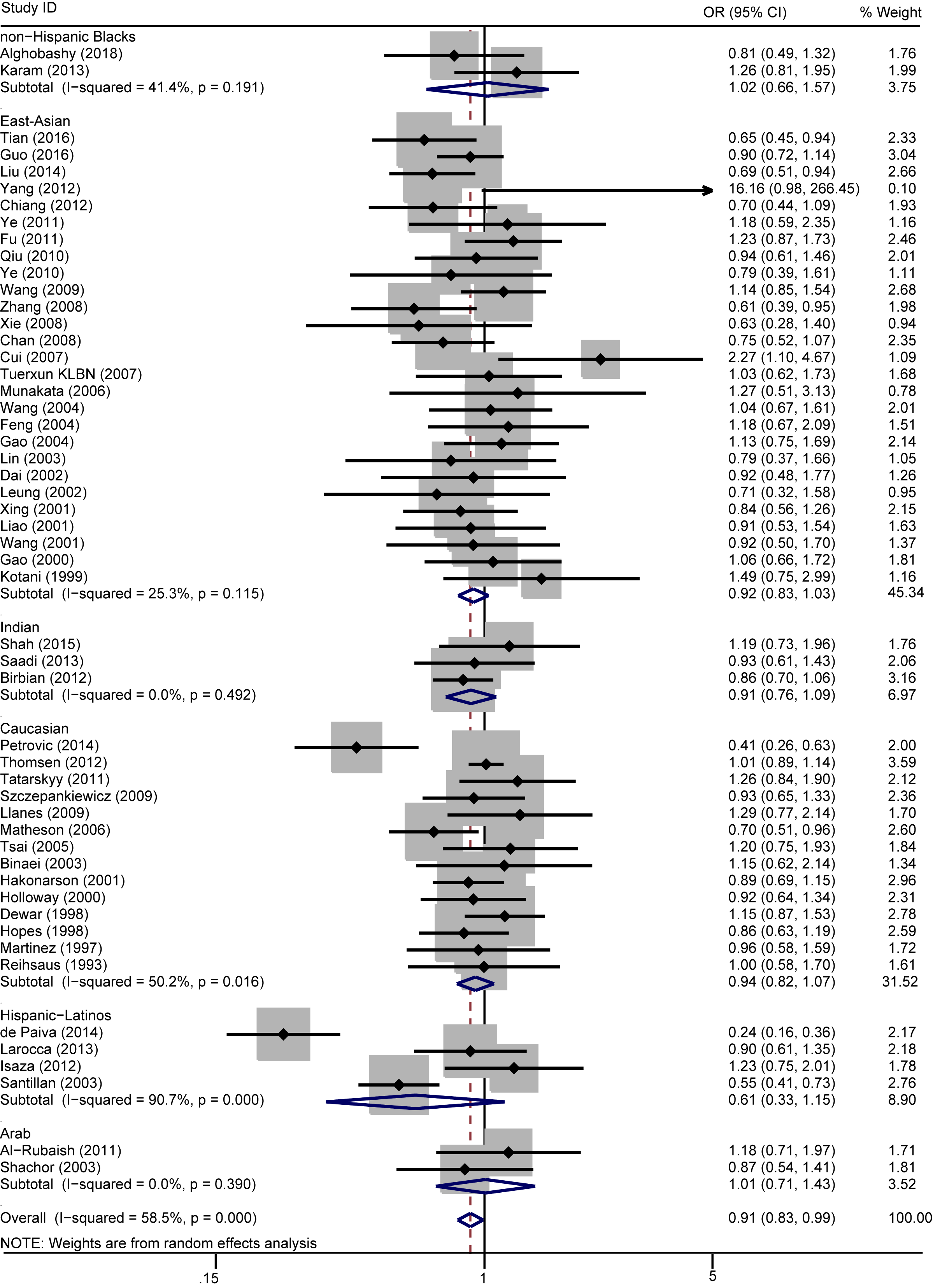

Supplement: Supplementary file 8 — Additional file 8: Figure S4. Forest plots of the association between the ADRB2 rs1042714 polymorphism and risk of asthma in the allele comparison. G vs. C allele. Each study is shown by an OR estimate with the corresponding 95% CIs. The horizontal lines denote the 95% CIs and the squares represent the point OR estimate of each study. The size of the square is proportional to its inverse-variance weight in the meta-analysis. The diamond represents the pooled meta-analysis effect size estimate. The stratified meta-analysis was performed regarding the ethnicity. (TIF 23295 kb) [file 12890_2019_962_MOESM8_ESM.tif]

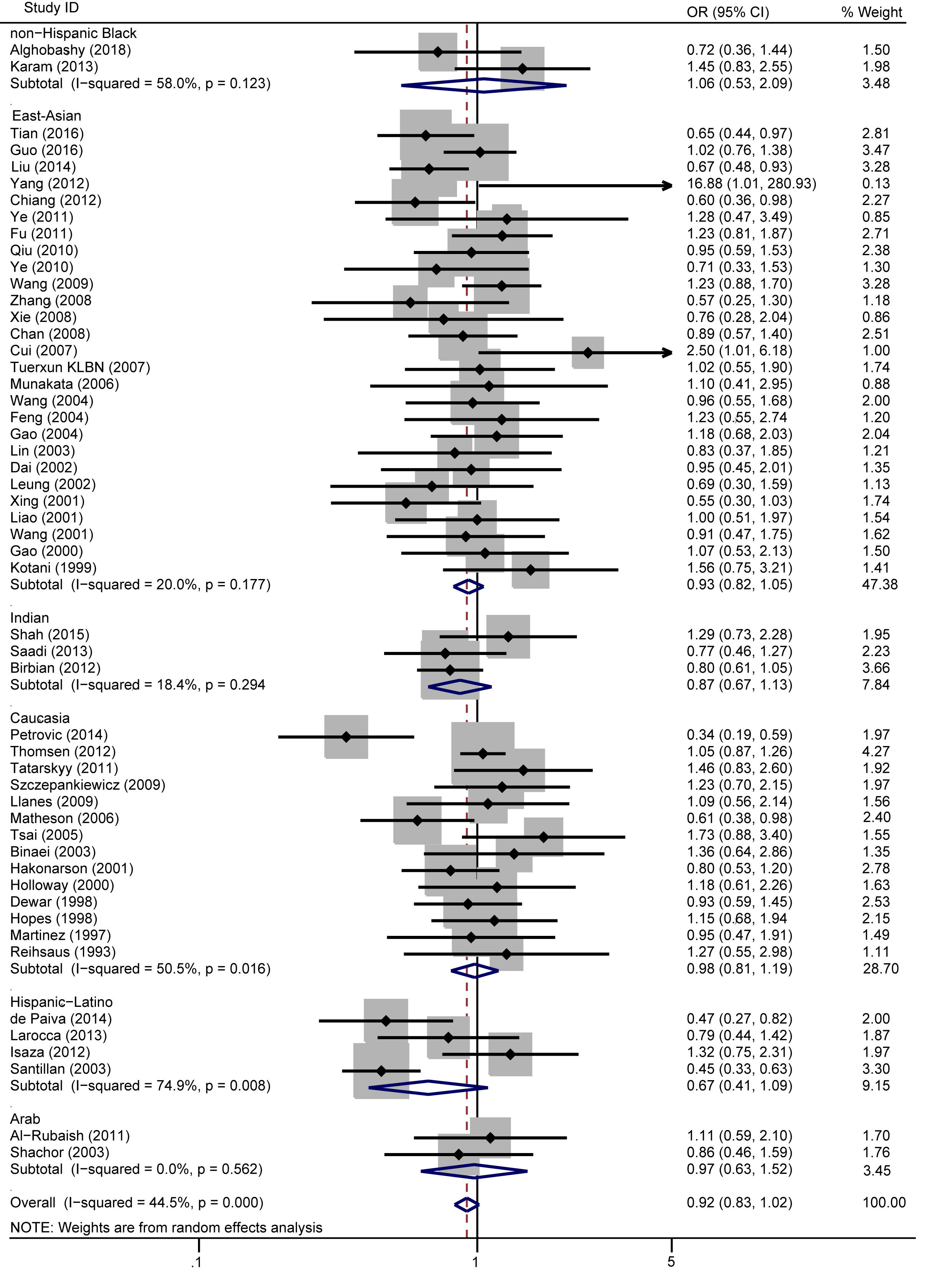

Supplement: Supplementary file 9 — Additional file 9: Figure S5. Forest plots of the association between the ADRB2 rs1042714 polymorphism and risk of asthma in dominant model comparison. GG + CG vs. CC genotype. Each study is shown by an OR estimate with the corresponding 95% CIs. The horizontal lines denote the 95% CIs and the squares represent the point OR estimate of each study. The size of the square is proportional to its inverse-variance weight in the meta-analysis. The diamond represents the pooled meta-analysis effect size estimate. The stratified meta-analysis was performed regarding the age. (TIF 24131 kb) [file 12890_2019_962_MOESM9_ESM.tif]
